# Australian clinical feasibility considerations for treatment of PTSD with cannabinoid-augmented exposure therapy

**DOI:** 10.1177/00048674231216587

**Published:** 2023-12-07

**Authors:** Luke J Ney, Wole Akosile, Christopher G Davey, Louise Pitcher, Kim Felmingham, Leah Mayo, Matthew Hill, Esben Strodl

**Affiliations:** 1 School of Psychology and Counselling, Faculty of Health, Queensland University of Technology, Brisbane, QLD, Australia; 2 Greater Brisbane Clinical School, Faculty of Medicine, The University of Queensland, Brisbane, QLD, Australia; 3 Department of Psychiatry, The University of Melbourne, Melbourne, VIC, Australia; 4 Trauma Strong, Brisbane, QLD, Australia; 5 School of Psychological Sciences, Department of Medicine, Dentistry and Health Sciences, The University of Melbourne, Parkville, VIC, Australia; 6 Department of Psychiatry and Hotchkiss Brain Institute, Mathison Centre for Mental Health Research & Education, University of Calgary, Calgary, AB, Canada

Posttraumatic stress disorder (PTSD) is a serious mental health condition that can be highly debilitating and represents a significant health challenge to the Australian community. As a disorder characterised by memory disturbances, avoidance, hyperarousal, as well as negative cognitions and mood, PTSD is usually treated with psychotherapy, with exposure therapy best supported by the available scientific literature. Underlying successful exposure therapy is the process of fear extinction, where the fear memory is overridden by a new safety memory which competes with and inhibits the fear memories of trauma. However, patients can experience high rates of treatment resistance and will often relapse following successful treatment.

Emerging evidence suggests that augmentation of the endogenous cannabinoid (endocannabinoid) system using medicinal cannabis is a potential method of improving PTSD treatment outcomes (Rehman et al., 2021). This hypothesis is supported by a plethora of preclinical data, as well as anecdotal reports of efficacy and growing human experimental work. However, all of this basic science is underpinned by the premise that extinction memory can be improved by medicinal cannabis through activation of the endocannabinoid system, in particular cannabinoid receptor 1. This work highlights the potential of cannabinoid augmentation during exposure therapy (i.e. clinical extinction training) can improve treatment outcomes in PTSD. However, it is critical to note that cannabinoid administration by itself is not sufficient to reduce preclinical fear, and that concurrent extinction training is necessary for remission from PTSD-like symptomology (Morena et al., 2018). Indeed, clinical trials of ad libitum cannabis administration show no efficacy for reducing PTSD symptomology (Bonn-Miller et al., 2021), and cross-sectional, anecdotal and retrospective data are mixed (Rehman et al., 2021). Therefore, by all arguments, cannabis might only improve PTSD treatment outcomes when used in conjunction with therapies that involve extinction training.

Despite the overall enthusiasm in the research community for this therapy combination, the clinical reality of such an approach is logistically challenging in ways that need to be systematically addressed in clinical trials and feasibility studies. First, while cannabis is anxiolytic in most users, it can be anxiogenic in some cases. Populations that are vulnerable to anxiogenic effects are those with contraindicating mental health – such as anxiety – and substance use disorders, both of which are frequently comorbid with PTSD. Therefore, the first noteworthy challenge to implementing cannabis-assisted exposure therapy is the risk that patients may experience higher levels of distress during therapy, which may impact treatment engagement and increase dropout rates – which for exposure therapies are already quite high. As a matter of priority, future clinical trials should test the feasibility and tolerability of the combination of these therapies through careful analysis of patient and clinician feedback.

In terms of drug classification and drug effects, cannabis sits somewhere in the spectrum between psychedelics and depressants, though it can also be classified as a stimulant. One of the challenges of psychedelic therapies – including 3,4-methylenedioxymethamphetamine (MDMA) – is that longer therapy session times and multiple attending clinicians are usually required. These logistical challenges make psychedelic therapies an expensive option that cannot be practically considered as a first-line approach for most patients, and these limitations may be at least partially shared with medicinal cannabis (Boehnke et al., 2022). The amount of clinician time needed to support a patient using medicinal cannabis concurrently with exposure therapy is not currently clear, and since this specific combination of treatments has not been trialled, it is unclear whether this approach will require specialised clinical spaces, like with MDMA, or whether it will be able to used with existing infrastructure. Future clinical trials need to establish the exact clinical parameters that enable safe and successful treatment of PTSD, though those of us working in this area are optimistic that the requirements of therapy will be less intensive compared to other psychedelics (e.g. ketamine, MDMA and psilocybin). More broadly, detailed and inclusive clinical governance protocols are needed for treatments seeking to involve vulnerable patients (e.g. with PTSD) under the influence of any mind-altering substance while undergoing clinical treatment.

However, an issue that cannot be ignored in Australia is the restrictions on driving for those who are using any kind of product – medicinal or not – containing delta-tetrahydrocannabinol (THC). Although rules vary across states, in most states, no one is permitted to drive with detectable levels of THC in their system. This means anyone undergoing cannabis-based therapies is not able to drive for the entirety of their treatment, including an additional (approximately) 1 week following treatment cessation, due to the long and highly variable wash-out period of detectable THC in the peripheral nervous system. This means that exposure therapy treatments should be ideally condensed to as short a time as possible. In Australia, patients receive Medicare rebates for up to 10 sessions of psychotherapy conducted by registered allied health practitioners, so the default for clinical outpatient practice is to do one session of exposure therapy per week, for 10 weeks. However, evidence suggests that intensive exposure therapy over a shorter period is just as effective as the same number of sessions over a longer period (Dell et al., 2023), suggesting that it may be feasible to administered a cannabis-augmented exposure therapy treatment over 4 or less weeks. While this is highly amenable to inpatient scenarios, standardisation of treatment protocols for outpatients over an intensive period will need to be achieved. However, this is an issue specific to Australia and may change with changes to legislation surrounding cannabis use and driving. A more pressing challenge in private practice is that the Australian Medicare rebates are only for ten 50-minute sessions, which could severely impact the feasibility of longer treatment protocols involving prolonged exposure as well as cannabis-adjunct exposure therapy that will likely require longer patient monitoring by clinical staff.

Finally, exposure therapy sessions are not always positive for a patient with PTSD. If medicinal cannabis is a general memory enhancer under stress and a patient does not have a constructive session, cannabis use prior to that session may inadvertently consolidate memories that do not support recovery from PTSD. Although this possibility has not been tested, it suggests that cannabis administration prior to exposure therapy may not be the best option, and a dosing schedule that allows the therapist to selectively enhance successful extinction learning using cannabis at their discretion would produce the best treatment outcomes. Clinical trials need to test whether consistent pre-therapy dosing reduces the efficacy of treatment; however, there is evidence that suggests that within-session affect may not predict eventual exposure therapy outcome. Moreover, dose timing with respect to anticipated pharmacokinetics – which depend on the route of administration – needs to be carefully aligned with the beginning of the therapy session. This will most likely require a route of administration that results in rapid peak plasma concentrations, though what type of route is best for therapy is still unclear. Again, these issues cannot be solved simply by theorising and need to be systematically tested in clinical trials testing feasibility and efficacy of different treatment approaches.

In conclusion, although the academic and public communities are interested in the potential of improving treatment of PTSD with medicinal cannabis, we argue that multiple practical and logistical challenges remain to be solved. The disadvantage of not considering these issues in clinical trials is that trialled treatment approaches may not be feasible for patients and may reduce the efficacy and disseminability of the treatment overall, for example, using the wrong route of administration with the wrong timing. Treatment protocols should be trialled and adjusted based on careful consideration of patient and clinician feedback to ensure that cannabis augmented exposure therapy of PTSD is feasible and effective.
